# Reduced Polymorphism in Domains Involved in Protein-Protein Interactions

**DOI:** 10.1371/journal.pone.0034503

**Published:** 2012-04-03

**Authors:** Zohar Itzhaki, Hanah Margalit

**Affiliations:** Department of Microbiology and Molecular Genetics, IMRIC, Faculty of Medicine, The Hebrew University of Jerusalem, Jerusalem, Israel; National Institutes of Health, United States of America

## Abstract

Genome sequencing of various individuals or isolates of the same species allows studying the polymorphism level of specific proteins and protein domains. Here we ask whether domains that are known to be involved in mediating protein-protein interactions show lower polymorphism than other domains. To this end we take advantage of a recent genome sequence dataset of 39 *Saccahromyces cerevisiae* strains and the experimentally determined protein interaction network of the laboratory strain. We analyze the polymorphism in domain residues involved in interactions at various levels of resolution, depending on their likelihood to be interaction mediators. We find that domains involved in interactions are less polymorphic than other domains. Furthermore, as the likelihood of a residue to be involved in interaction increases, its polymorphism decreases. Our results suggest that purifying selection operates on domains capable of mediating protein interactions to maintain their function.

## Introduction

Most proteins are composed of domains, which are their functional and structural units. As such, domains are expected to be less prone to diverge than extra-domain regions. Indeed it was shown that domains are conserved between species [1], [2] and that critical functional residues within them are conserved [3]–[6]. Recently, using genome sequence data of 28 strains of *Saccharomyces paradoxus*, Vishnoi *et al.* demonstrated that residues within domains are less polymorphic than residues outside the domains [7].

It is widely established that domain-domain interactions play a major role in interactome networks [8], [9]. Furthermore, using large-scale data of protein-protein interactions (PPIs) and structural data of protein complexes it was shown that there is a limited set of domains that are exploited for mediating PPIs [9]–[15], and that different organisms use the same domain-pairs for mediating their PPIs [16]–[18]. This suggests that interacting domains would be tightly conserved, more than other protein domains. To address this question, reliable data of protein orthologous relationships in different organisms are needed, as well as data of interactome networks of the studied organisms. Alternatively, this question can be addressed by analyzing polymorphism in sequence data of very close organisms of the same species, where the PPI networks can be projected from one organism for which such data are available to the others. Fortunately, Liti *et al.* have recently provided genome sequence data of 39 strains of *Saccharomyces cerevisiae* [19]. These data enable the analysis of polymorphism in domains involved in PPIs, assuming that the widely studied PPI network of the *S. cerevisiae* laboratory strain is shared by all other strains.

## Results and Discussion

**Figure 1:**
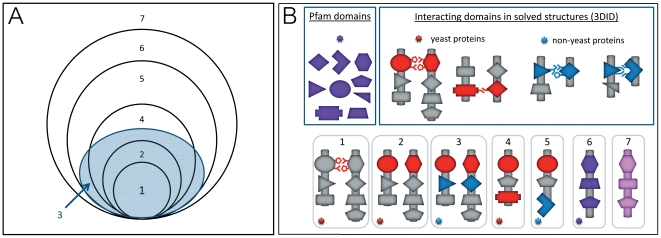
Residues included in the analysis at the different resolution levels. We focused on seven resolution levels of residues/codons within *S. cerevisiae* genes: 1) Structurally-determined interacting residues - the residues/codons involved in protein interactions, as reported in the 3DID database based on *S. cerevisiae* complexes solved by crystallography [9]. 2) Structurally-determined interacting domains - All residues/codons in yeast domains that were shown to mediate interaction in PPI structures solved by crystallography. 3) Residues in yeast domains inferred as mediating interactions - All residues/codons in domain-pairs in yeast PPIs, capable of mediating interaction. These domain-pairs were shown by crystallography to mediate PPIs in solved complex structures (not necessarily in yeast) and were projected onto yeast PPIs (Methods). 4) Inferred interaction-mediating domains in yeast - All residues/codons in domains (not pairs) that were found in solved structures as capable of mediating PPIs in yeast. These include domains in proteins involved in PPIs and domains in proteins that were not yet shown to be involved in PPIs. 5) Inferred interaction-mediating domains - All residues/codons in domains (not domain-pairs) that were found in solved structures as involved in PPIs, not necessarily in yeast. 6) Domains - All residues/codons in domains (as opposed to extra-domain residues. 7) Proteins - Whole-protein residues (domains and extra-domains). (**A**) A schematic diagram describing the resolution levels. The figure is not scaled. (**B**) Schematic representation of the residues included in each resolution levels. The up panel describes the source of domains, interacting domains and interacting residues. The bottom panel shows schematically the residues/domains included in each resolution level and indicates the data source this classification is based on by the star color: red - data based on interacting domains in yeast proteins; blue - data based on interacting domains in other organisms; purple - data based on Pfam domains; pink - extra-domain regions.

**Figure 2:**
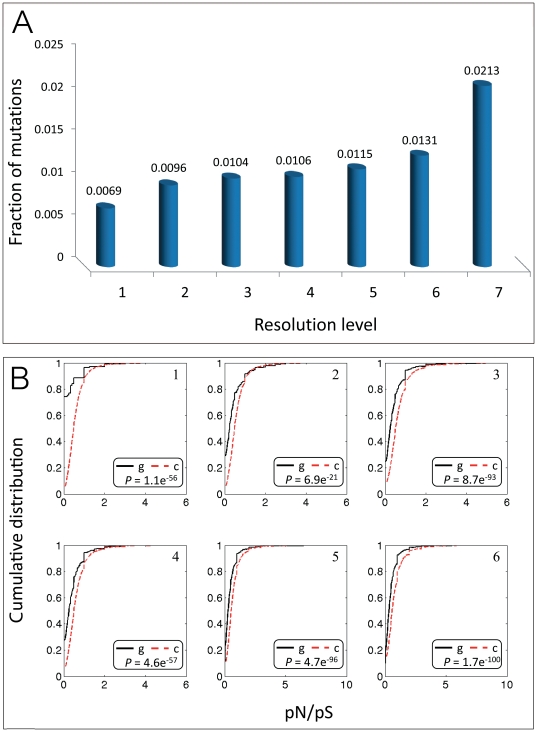
Analysis of residue conservation in interacting domains. (**A**) Average fractions of non-synonymous mutations in residues determined by the various resolution levels (see caption of Figure 1). A non-synonymous mutation was determined if there was a substitution in at least one strain, compared to the laboratory strain. (**B**) Comparison of pN/pS values between residues in a set determined by a resolution level and a complementary set of residues: 1) Interacting residues in yeast proteins based on solved structures were compared to non-interacting residues. 2) Yeast interacting domains based on solved structures were compared to non-interacting domains. 3) Domain-pairs in PPIs that can be mapped to structurally solved domain-domain interactions were compared to the other domains. 4) Domains documented in yeast as interacting were compared to domains that were not documented as interacting in solved structures in yeast. 5) Domains that were documented in yeast and other organisms as interacting domains were compared to domains that were not documented as interacting in solved structures in any organism. 6) Residues in protein domains were compared to residues that do not reside within domains (extra-domain regions). These six groups correspond to groups 1–6 in Figure 1. g: residues in studied set. c: residues in complementary set. P-values of Kolmogorov-Smirnov test (applying FDR correction) are given at the bottom of each panel.

We analyze the polymorphism in *S. cerevisiae* residues in domains associated with PPIs (Figure 1). The data we used contain the gene and protein sequences of 39 *S. cerevisiae* strains included in the *Saccharomyces* Genome Resequencing Project database [19]. These strains were collected from around the world and their genomes were sequenced by ABI shotgun sequencing and Illumina GA (Solexa). The database supplies all single nucleotide polymorphisms (SNPs) in the 38 strains compared to the genome of the reference laboratory strain. In addition, it supplies multiple sequence alignments of the protein sequences of the 39 strains. We used these data to investigate synonymous and non-synonymous SNPs in domain residues involved in protein interactions. The number of solved structures of *S. cerevisiae* complexes is limited, and consequently the dataset of *S. cerevisiae* domains that were reliably determined as involved in protein interactions is small. Therefore, we extended the dataset of interacting domains by inferring about domain interaction from other data sources, which differed in the reliability that could be attributed to the interaction. We use the term ‘levels of resolution’ to refer to the classification of residues as interacting and non-interacting at different reliability levels. Our analysis included seven levels of resolution, as defined in Figure 1 (see also Methods). Only proteins with Pfam domain annotations [20] that had at least one SNP were included in the analysis, resulting in a dataset of 3,927 proteins. This dataset was further reduced to 3,669 proteins after filtering alternative splicing variants and ambiguously annotated proteins. For each resolution level we calculated the fractions of the non-synonymous SNPs out of all relevant residues of a protein, and then computed the average of these fractions (Methods). As shown in Figure 2A, the fraction of non-synonymous SNPs increases as the reliability that these residues are involved in protein interactions decreases. We repeated the analysis using different thresholds for determination of SNPs (Supporting Figure S1A and Methods). The trend of reliable interacting residues being less polymorphic persisted for all thresholds.

As it was previously reported that highly expressed proteins evolve slower than weakly expressed ones [21], we turned to verify that our results are not affected by variation in expression levels. To this end we repeated our analysis when the proteins were divided to two groups by their expression levels [22]: weakly-expressed proteins and highly-expressed proteins (Methods). For both groups we found reduced polymorphism in interaction-mediating domains (Supporting Figure S2), consistent with the above results and emphasizing the robustness of our findings.

Due to the specific population structure of the yeast strains that were sequenced and in order to cope with the effect that it might have on our analysis, we repeated the analysis with representatives of the various clades. The selection of these representatives was done iteratively using a tree of all strains, built based on SNP differences in their sequences [19]. In each iteration the selected strain was the most distant one from the other already selected strains. The results using six and ten representative strains were consistent with the aforementioned results, further supporting our conclusions (Supporting Figure S1B,C).

To further investigate the conservation of interacting domains taking into account the local mutation rate, we calculated the non-synonymous to synonymous mutation ratios (pN/pS). This analysis was carried out for the residues at various resolution levels as in the previous analyses, each time comparing the distribution of pN/pS ratios of the relevant residues in the studied proteins to their distribution in a complementary set of residues in the same proteins (Figure 2B). This comparison revealed that the pN/pS values of interacting domains are lower than those of non-interacting domains, implying that residues in interacting domains are more conserved. We repeated the analysis using different thresholds for SNP determination, as described above, and found that the phenomenon is consistent (Supporting Figure S3). We verified that our results are not biased due to over-representation of specific paralogs that may have specific conservation patterns, using the dataset of Wapinski *et al.* of paralogous proteins in *S.cerevisiae* [23]. We kept a representative protein for each paralogous cluster and repeated the analysis, obtaining results consistent with the above.

Next, we investigated the substitutions of amino acids in the 38 *S. cerevisiae* strains compared to the laboratory strain. We compared the distribution of the substitution scores between interacting and non-interacting residues, defined for different resolution levels as in Figure 2B (see Methods). We found that all comparisons (except for the comparison of the residue set in the highest resolution level to its complementary set, which was based on a small number of scores) showed statistically significant differences (p-values ranged between 9×10^−3^ and 4.6×10^−34^, applying FDR correction). In all the comparisons the residues in interacting domains have substitutions with higher scores than the residues in the non-interacting domains, implying that they are substituted by similar amino acids that probably preserve their functionality.

Our analysis provides a bird's eye view on the polymorphism in yeast residues involved in protein interactions at various levels of resolution. To obtain a more concrete understanding of our results, we provide as an example a closer look at one protein in our data, DCP1, mRNA-decapping enzyme subunit 1 (Q12517), a single domain protein whose homodimer structure was solved by crystallography [24] and included in the 3DID database. The domain mediating the homodimerization is PF06058 (resolution 2 in our analysis) and 18 specific residues were determined as participating in the interaction (resolution 1 in our analysis). Analyzing the multiple sequence alignment and SNPs regarding residues at resolution level 1, we obtain that all interacting residues exhibit no polymorphism. At the domain resolution (resolution 2) we identified three positions with non-synonymous substitutions. Thus, interacting residues of this domain are less polymorphic, consistent with the trend observed for the whole database. The crystal structure and the polymorphism results suggest that the specific interacting residues have a greater effect on the stability of the complex than other domain residues. To substantiate this conjecture we applied to DCP1 dimer the FoldX algorithm [25], [26], an algorithm that quantitatively estimates the importance and contribution of interface residues to the stability of a protein complex. This algorithm performs a computational alanine-scan for residues in a protein interface and calculates the change in the energy of the complex. Application of the algorithm to 16 residues in the interface that are classified as interacting revealed an average energy change of 0.94 kcal/mole per residue, alanine substitution of a polymorphic non-interacting residue of the domain was predicted to even increase the complex stability (−2.15 kcal/mole), and substitutions in seven non-interacting, non-polymorphic interface residues were predicted to destabilize the complex by an average of 0.17 kcal/mole per residue. These results are consistent with the expectations from the SNP analysis and complex structure, where substitutions of non-polymorphic domain residues have on average a greater effect on complex stability than the substitution of the polymorphic residue (0.17 versus −2.15 kcal/mole), and the non-polymorphic interacting residues have a greater effect on complex stability than non-polymorphic non-interacting residues (0.94 versus 0.17 kcal/mole).

In summary, our study shows at the different resolutions that residues in domains associated with PPIs are less polymorphic than in other domains. At the lowest resolution level, our results are consistent with that of Vishnoi *et al.* [7], who found that residues in domains are less polymorphic than extra-domain residues. At a higher resolution are domains that were found in yeast and other organisms to mediate PPIs. We found that their residues are less polymorphic than residues in other domains. This further emphasizes the functionality of these domains. These domains were suggested to constitute a limited repertoire of domain-pairs that play a role as PPI mediators, and their lower polymorphism among yeast strains is consistent with this supposition. At the finest resolution, of yeast protein residues that are included in interacting domains or were shown to be involved in interaction in crystal structures, our results imply that these residues and domains undergo tighter selection to preserve their functionality.

## Methods

### Fractions of non-synonymous SNPs

We determined positions with non-synonymous substitutions in comparison to the laboratory strain as those in which there was a different amino acid in at least a pre-set number of strains (n). The analysis was repeated for n ranging from 1 to 37. We counted one SNP per a position even if the substitutions differed between the various strains. In each such analysis we counted the fraction of polymorphic positions out of all relevant positions of a protein, and computed the average of these fractions over all proteins in our data.

### Projection of interacting domain-pairs onto PPIs

Each protein in our data was labeled by its domains according to Pfam database [2]. Following our procedure [16] we used the 3DID database [9] as a source for structurally-determined interacting domain-pairs. Two interacting proteins were predicted to interact through two particular domains if one of them included one domain and the other included its interacting partner, as recorded in the 3DID database.

### Protein expression data

We used data of yeast protein expression in log-phase growth from Ghaemmaghami *et al.* [22], and defined two groups of proteins according to their expression: weakly expressed proteins – with less than 40 copies per cell (a total of 958 proteins), and highly expressed proteins – with more than 1,000 copies per cell (a total of 2,006 proteins).

### Amino-acid substitution analysis

In order to examine the substitutions of amino acids in the 38 *Saccharomyces cerevisiae* strains compared to the laboratory strain, we used the Blosum100 substitution matrix, which is suitable for pairwise comparisons of closely related proteins [27], and retrieved a substitution score for each of the mutated amino acids. We then compared the distributions of substitution scores between interacting and non-interacting residues, defined for different resolution levels as in Figure 2B. Comparison of the distributions was carried out using Kolmogorov-Smirnov test, applying FDR correction.

## Supporting Information

Figure S1 Fraction of non-synonymous mutations in each resolution level, using thresholds for SNP determination (*i.e.* a position is determined as having a SNP if a mutation occurs in a number of strains that exceeds the threshold).
(DOCX)

Figure S2 Analysis of residue conservation in interacting domains in highly and weakly expressed proteins. Average fractions of non-synonymous mutations in residues determined by the various resolution levels.
(DOCX)

Figure S3 Heat map of the p-values of Kolmogorov-smirnov tests comparing pN/pS between a set of residues defined by a resolution level and a complementary set, when mutations are determined by different thresholds, as in Figure S1.
(DOCX)
