# Supplementary material for: Reduced Polymorphism in Domains Involved in Protein-Protein Interactions
Source: PLoS One. 2012 Apr 3;7(4):e34503. doi: 10.1371/journal.pone.0034503 (PMC3317993; doi:10.1371/journal.pone.0034503)
Supplement: Figure S1 — Fraction of non-synonymous mutations in each resolution level, using thresholds for SNP determination (i.e. a position is determined as having a SNP if a mutation occurs in a number of strains that exceeds the threshold). (DOCX) [file pone.0034503.s001.docx]

Figure S1

(A)


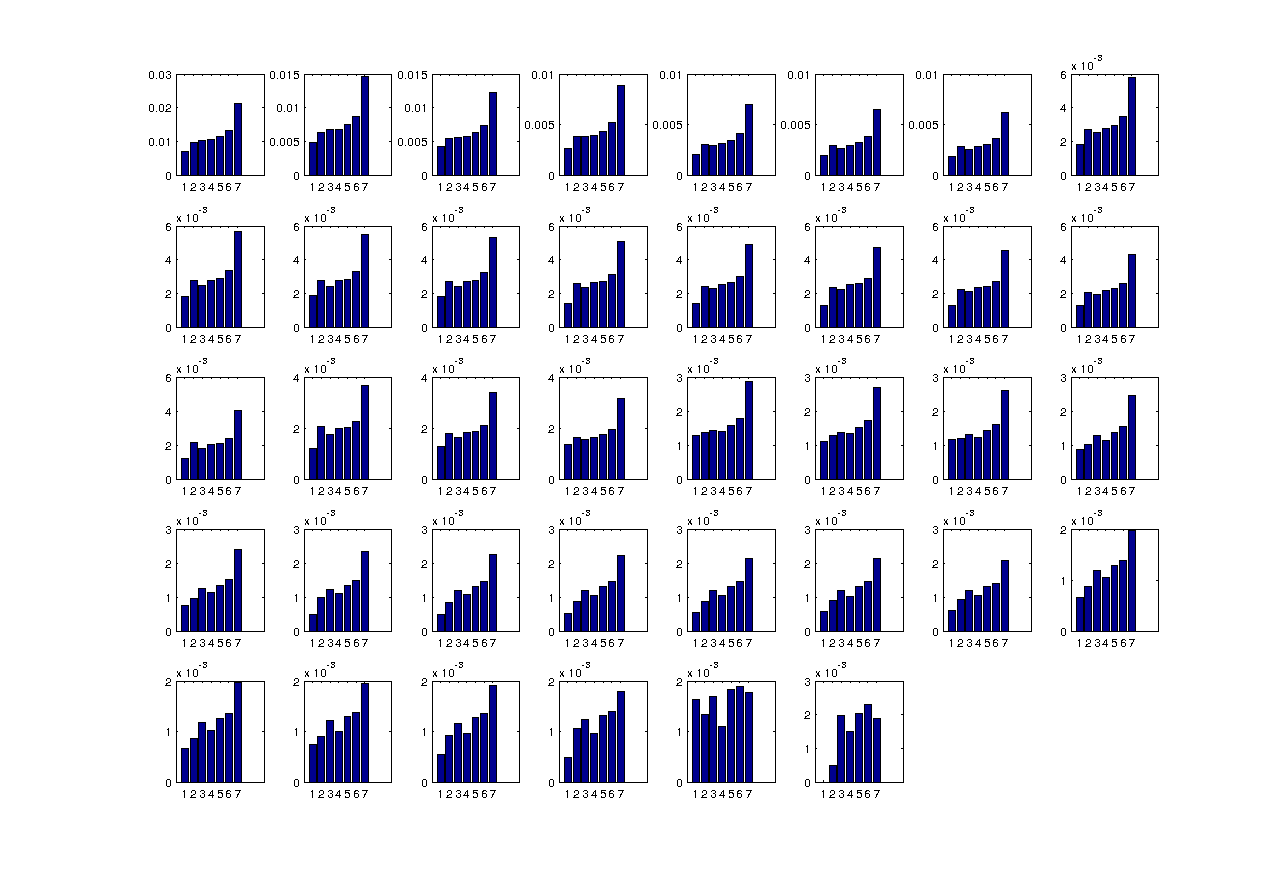


Fraction of non-synonymous mutations

(B)

Fraction of non-synonymous mutations


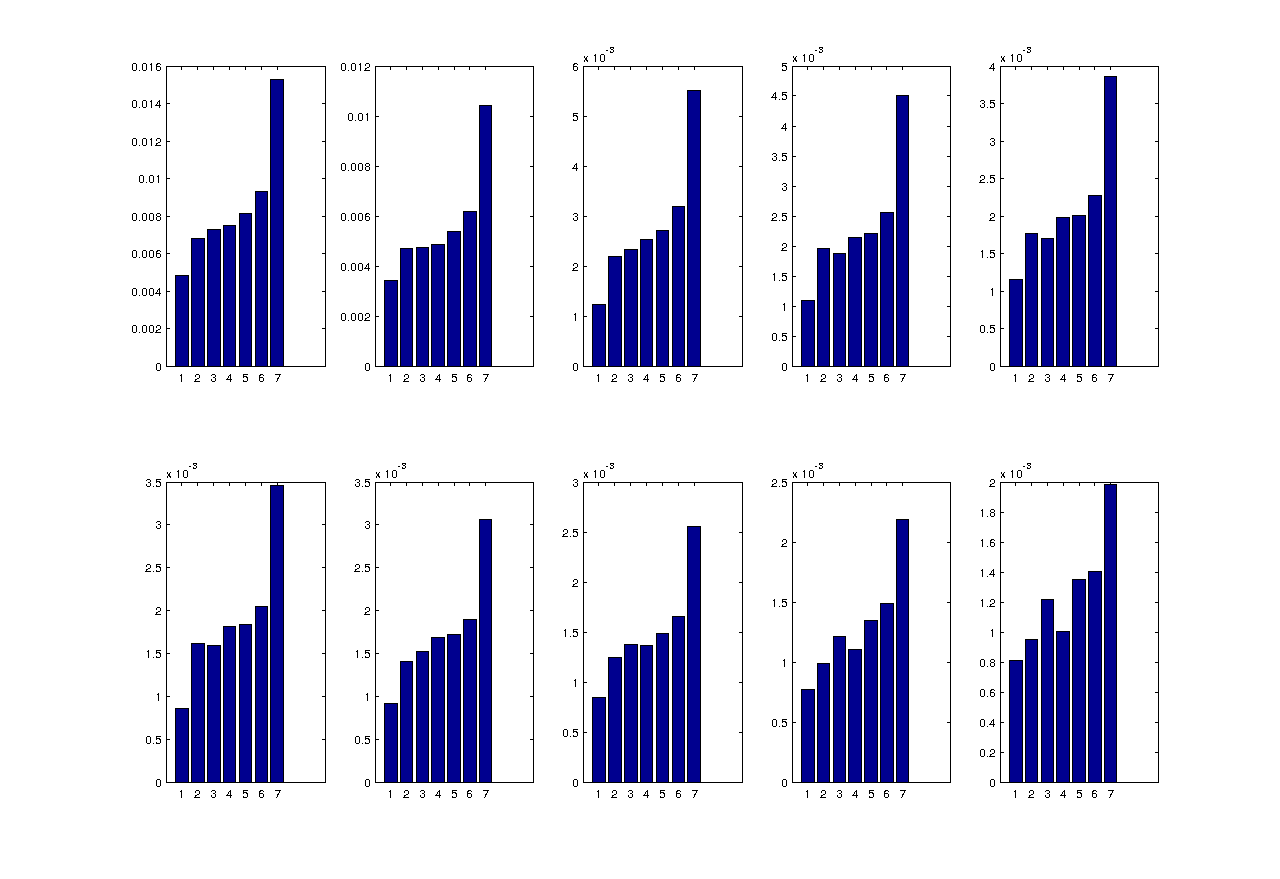


(C)


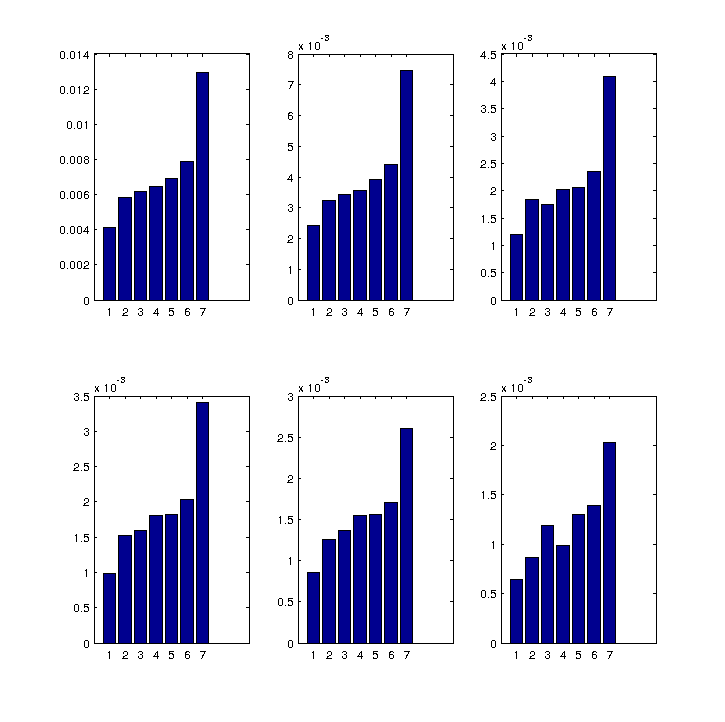


Fraction of non-synonymous mutations

Fraction of non-synonymous mutations in each resolution level, using thresholds for SNP determination (*i.e*. a position is determined as having a SNP if a mutation occurs in a number of strains that exceeds the threshold). Only proteins with at least one position that is determined as having a SNP according to the relevant threshold were taken into consideration.

Resolution levels are as in the main text: (1) Residues/codons involved in the protein interactions. (2) All residues/codons in yeast domains that are involved in the protein interactions. (3) Residues/codons in domain-pairs inferred from other solved structures. (4) Residues/codons in domains documented in yeast as interacting. (5) Residues/codons in domains that were documented in yeast and other organisms as interacting domains (6) Residues/codons in domains, and (7) Residues/codons in whole proteins.

**(A)** Using all 39 strains. **(B)** Using as representatives the ten most distant strains. **(C)** Using as representatives the six most distant strains.

Axes: X-axis – the seven resolution levels. Y-axis – fraction of non-synonymous mutations in the residues defined by the resolution level (not uniformly scaled).

Each panel from left to right represents an increasing threshold. *i.e:* the upper left panel represents the results using the threshold of 1; the second panel represents the results using the threshold of 2, etc.
