# Supplementary material for: Reduced Polymorphism in Domains Involved in Protein-Protein Interactions
Source: PLoS One. 2012 Apr 3;7(4):e34503. doi: 10.1371/journal.pone.0034503 (PMC3317993; doi:10.1371/journal.pone.0034503)
Supplement: Figure S2 — Analysis of residue conservation in interacting domains in highly and weakly expressed proteins. Average fractions of non-synonymous mutations in residues determined by the various resolution levels. (DOCX) [file pone.0034503.s002.docx]

Figure S2

Fraction of mutations

Resolution level

A

Fraction of mutations

Resolution level

B

Analysis of residue conservation in interacting domains in highly and weakly expressed proteins

Average fractions of non-synonymous mutations in residues determined by the various resolution levels (see caption of Figure 1). **(A)** Highly-expressed proteins. **(B)** Weakly-expressed proteins.
