# Supplementary material for: Reduced Polymorphism in Domains Involved in Protein-Protein Interactions
Source: PLoS One. 2012 Apr 3;7(4):e34503. doi: 10.1371/journal.pone.0034503 (PMC3317993; doi:10.1371/journal.pone.0034503)
Supplement: Figure S3 — Heat map of the p-values of Kolmogorov-smirnov tests comparing pN/pS between a set of residues defined by a resolution level and a complementary set, when mutations are determined by different thresholds, as in Figure S1. (DOCX) [file pone.0034503.s003.docx]

Figure S3


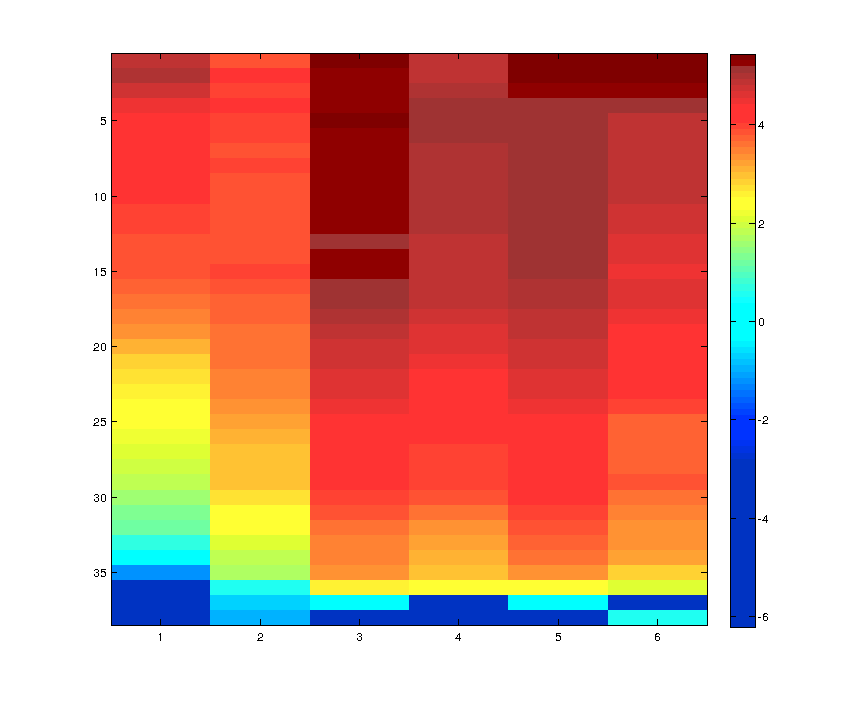


Threshold

-log(p)

Resolution level

Heat map of the p-values of Kolmogorov-smirnov tests comparing pN/pS between a set of residues defined by a resolution level and a complementary set, when mutations are determined by different thresholds, as in Figure S1. (1) Interacting residues in yeast proteins based on solved structures were compared to residues that were not shown to interact. (2) Residues in yeast interacting domains based on solved structures were compared to residues in domains that were not shown to interact. (3) Residues in domain-pairs in PPIs that can be mapped to structurally solved DDIs were compared to residues in other domains (4) Residues in domains documented in yeast as interacting were compared to residues in domains that were not documented as interacting in solved structures in yeast. (5) Residues in domains that were documented in yeast and other organisms as interacting domains were compared to residues in domains that were not documented as interacting in solved structures in any organism. (6) Residues in protein domains were compared to residues that do not reside within domains (extra-domain regions). The values are of –log(p).
